# The Collection of Bills

**Published:** 1904-07

**Authors:** Leslie Barnes Boutwell


### THE COLLECTION OF BILLS.[1]

BY LESLIE BARNES BOUTWELL, D.M.D.

Mr. President and Fellow-Members of the Harvard Odontological Society,—Many years ago, at a district school which I attended as a small boy, there was an occasional morning set apart for declamation, or "speaking pieces," as we used to term it,

---

[1] Read before the Harvard Odontological Society, April 28, 1904.

and the favorite effort of the average badly scared youngster used to be a choice excerpt which began thus:

> "You'd scarce expect one of my age
> To speak in public on the stage."

I will spare you the description of how the knees would knock together, the cold sweat stand out upon the body, and the tongue and brain refuse their co-ordinate labor as the declaimer faced the combined stare of schoolmates, friends, relatives, and the occasional committeeman, and merely call your attention to the parallelism of the present case and that of the youngster alluded to. You will probably discover the analogy for yourselves as this effort continues.

It is useless to attempt to give you gentlemen any information from a dental point of view, and the choice of a subject is a most serious one to the poor unfortunate drawn as an essayist; and any essay in that direction would hardly instruct, though it probably would amuse you. When you go back mentally to that time in your early practice that you faced your first problem, in, for instance, trying to combat some carefully cherished fallacy in a patient, real or prospective, and endeavoring to make him believe that a perfect-fitting artistic denture cannot be made for five dollars, or a large contour gold filling inserted at the advertised price of one dollar, even though you carefully explain that the aforesaid attractive advertisement was not fathered by you,—in such cases they have a sincere belief in the old adage, "What man has done, man can do,"—such problems fade into insignificance beside the momentous one of what to talk to you about.

I have therefore chosen for a subject one that will at least interest you, and one on which from many years of business experience I can speak with some authority, and that is, the collection of bills, and in combining enough of business principles with our profession to render that collection easier and more systematic, the latter being, in my opinion, the great desideratum; and I might preface my few remarks with a text from the "good book" as follows: "And he took him by the throat, saying, Pay me that thou owest." Do not be alarmed because I have begun with a text. This sermon will hardly go beyond "Firstly, my brethren." Probably the creditor in the foregoing was a dentist who had been unfortunate enough to do two hundred pence worth of work for one hundred, and after much unsuccessful dunning was endeavoring to forcibly collect his little bill, and although in the present civilized age we can scarcely go to the extent of bodily punishment for delinquent debtors, yet there is so little change in human nature in 1900 years, that we sympathize with the man of old who resorted to extreme measures in collecting from a particularly exasperating case. You who have threshed out your wheat, and have gathered the kernels of experience from years of practice, have also, to a greater or less extent, so sifted that practice that you have comparatively little trouble in collecting; but to us who are starting—particularly those of us who have a fairly large transient practice—it is a serious question when, and when not, to give credit, and when credit is given and abused more or less, how far are we justified in pushing our claims?

From a twenty years' experience as credit man in commercial life, I incline to the radical course of "taking them by the throat" and collecting anyway—peaceably if possible, forcibly if necessary; and as to the various methods, steps, and gradations of appeal to the debtor before invoking the aid of the law, each professional man has his choice, but it is all a means to an end, as the debtor who requires urging to settle is truly an "absent-minded beggar," and, therefore, the burden of our song to him should be insistently, "pay—pay—pay." I believe that in ninety-nine cases out of a hundred the patient is honest and means to pay. The one-hundredth one must be dealt with rigorously, and usually a mild pacific course, such as intimating ever so slightly that the account will be placed in a lawyer's hands for collection if not paid before a certain time, is vastly insufficient. I find that a statement to the effect that the poor debtors' court will be the ultimate result if payment is not forthcoming at once usually makes an impression and brings about a state of mental disquietude, which ceases only when the bill is paid. You have given of your best, time, material, and, greatest of all, nervous energy, and your charge should be paid gratefully, but if after repeated duns payment is not made, then make up your mind that you would lose the patient anyway, and take such action as will secure the bill, lest eventually you lose both.

Of course, such cases are rare, happily, but they are like the poor, "always with us," and unfortunately there is more joy over collecting one such bill than over those of "ninety and nine just men." It is a problem you are constantly called upon to decide, and while we are urged to err if necessary upon the side of mercy, yet why should we be incessantly asked to extend mercy to those who have no mercy upon us,—the very ones, usually, who have taxed our nervous energy the most. I believe that there is a lack of simple business principle in most professional men, particularly in sending out bills and statements, regularly and impartially, to all patients, and which lack results in much monetary loss.

The condition of mind which enables a debtor to disregard the receipt of two or more bills will usually enable him to sleep peacefully, even though he eventually does not pay the bill at all, and for such cases the cure is a sharp, harsh remedy analogous to the surgeon's knife, rather than the bloodless method; and then again, if financial care is not possible, excision is the most conducive to your future comfort, as well as a lesson to the delinquent not to assume financial obligations too easily. It might be laid down as a cardinal principle, that a man who is offended at receiving a bill is not a desirable addition to one's list of patients.

You and I receive bills the first of the month (not often, we will hope), and esttle them as promptly. Why, then, should we give more credit than we ask? Understand me—it is the debtor who makes no response at all to bills, statements, and letters that I refer to; patients that eventually pay in full, even though but a little at a time, are desirable ones to most of us. It is the conscienceless one who remains silent to all appeals, after he has got his work finished with you, at whom these strictures are directed. To such I personally show not a particle of consideration, and have collected my bill in all cases of the kind (where the debtor has not defaulted) by carrying them to court, even though it cost me nearly the whole amount of the bill, as there are few who care to have the stigma of such a conclusion fixed upon them.

Among all the problems outside the actual practice of dentistry, but within its province, I regard collection of bills as the one requiring the most tact, persistency, and knowledge of human nature. It also necessitates the most delicate "touch" known to mankind,—that which touches the pocket-book.

Several members of our profession have said, apropos to following up deliquent debtors closely, that they did not care to go too far, for fear of incurring the ill-will of the debtor, and the consequent loss of not only his patronage, but the loss of the persons who might be influenced by his recommendation as future patients.

I argue that a man who is so lacking in common honesty that he will not pay his bills, is also dead to any generous feeling that he will hardly go out of his way to influence new patients to come to us, and the chances are that those he did recommend would be of the same stamp as himself.

There is no question but that we all wish to collect our bills promptly, but I find many dentists who, after sending a few statements and meeting no response, cease to urge payment, and my effort here to-night is to suggest the same persistency applied to such cases as would be given to a difficult case in office practice. Each instance may require a different method of procedure,—one case may be necessary to be dealt with severely, and the next merely necessitate a call, to bring about a settlement.

In a few cases I have found that frequent visits by a youthful collector armed with the bill, and sent to the man's place of business with instructions to present it and ask for payment audibly enough so that by-standers may hear, is wonderfully effective as a last resort. I do not advocate extreme measures. I do, however, advise frequent calls, as I find that method successful when all sorts of written appeals fail. At least, that is my experience. The proportion of beats is small, I am glad to say, for out of the first five hundred names in my own practice I find only ten uncollectible bills, and of those the largest was under twenty dollars. The number, however, would have been larger but for a general adherence to the importance of persistent dunning.

